# Reconstruction of Trauma Dynamics Due to Ligature Strangulation by Using a Dynamometer: A Technical Report

**DOI:** 10.7759/cureus.48982

**Published:** 2023-11-18

**Authors:** Matteo Antonio Sacco, Cristoforo Ricci, Gionata Fragomeni, Carlo Filippo Bonetta, Fabrizio Cordasco, Saverio Gualtieri, Berardo Cavalcanti, Pietrantonio Ricci, Isabella Aquila

**Affiliations:** 1 Institute of Legal Medicine, Department of Medical and Surgical Sciences, Magna Græcia University of Catanzaro, Catanzaro, ITA; 2 Department of Jurisprudence, Magna Græcia University of Catanzaro, Catanzaro, ITA; 3 Department of Forensic Medicine, Forensic Medical Office, Corso Mazzini Giuseppe, Cosenza, ITA

**Keywords:** murder, violence, strangulation, asphyxia, autopsy, forensic science

## Abstract

Asphyxiation caused by violence, particularly through ligature strangulation, necessitates the application of a force that is characterized by a point of application, direction, and intensity. These properties can be quantified through the use of a dynamometer, which is composed of a graduated scale and a spring. In this particular study, an experimental model utilizing a dynamometer was employed to aid in the diagnosis and analysis of the dynamics of violent trauma resulting from homicidal ligature strangulation. The experimental model was applied to an attempted murder case involving strangulation. The primary challenge in this case was to establish the attempted murder scientifically, as the offender claimed that there had been no intent to kill, but instead an attempt to frighten the victim. To prove his assertion, the assailant emphasized the absence of strangulation injuries on the victim's neck. To investigate, a crane scale dynamometer was fixed on a cable and placed on a manikin's neck. The potential measurable combinations with the dynamometer were then compared to witness accounts and the injuries found on the victim. The utilization of a dynamometer in our case permitted the diagnosis and verification of a trauma that was undoubtedly caused by violent asphyxiation via strangulation. The information yielded by the dynamometer was subsequently submitted as scientific evidence in Court, serving to substantiate the intent to commit homicide and substantiate the credibility of the victim's testimony.

## Introduction

Strength is a measurable quantity characterized by a vector that includes a point of application, direction, and intensity. There are various instruments available to measure the intensity of force, but the most commonly used device is the dynamometer. A dynamometer comprises a spring made of bronze or steel and a graduated scale. When subjected to stress, the spring undergoes reversible variations as it acts like an elastic body. The graduated scale runs parallel to the vector and the deformation direction of the spring. The dynamometer works on the principle of Hooke's law, which states that the deformation of an elastic material is directly proportional to the amount of force that is exerted. Additionally, according to the formula F=K x ∆L, the elongation and applied force of an elastic body are directly proportional to each other, where K is a proportionality constant, F is the force vector, and ∆L is the deformation of the spring [1-3].

Typically, the lower portion of a dynamometer is furnished with a steel hook that is intended for the purpose of attaching an object to be weighed. When the body is placed on the hook, it deforms the spring due to the force of gravity, causing it to stretch to a specific extent which is depicted on a graduated scale. In the clinical realm, the dynamometer is a useful tool, particularly in the field of physical and neurorehabilitation, to appraise deficiencies in limb strength and monitor the advancement of functional recovery [4-5].

As of now, however, there is no documented research in the literature that discusses the use of the dynamometer for diagnostic purposes in the forensic field. In the context of investigating attempted murder by strangulation, an analysis of force may be necessary to measure the level of applied tension. Using a dynamometer attached to a fixed point, an operator can simulate the direction and force of traction, allowing for the replication of the dynamics of strangulation with potential homicidal instruments such as cables or ropes. Our experiment utilized this property of the dynamometer in a model of tying strangulation. By reproducing tractions of varying directions, we were able to compare the injuries sustained on a compressible manikin with gradually increasing strength values. This also allowed us to calculate the amount of force applied to the neck, including the victim's defensive reactions, based on differing versions of events given by both attacker and victim. This experimental model, combined with injury analysis and witness testimony, could prove invaluable in diagnosing an attempted murder by ligature strangulation.

## Technical report

This report concerns a case of a ligature strangulation attack. According to the victim's statement to the Judicial Authority, her spouse attempted to kill her by fastening an electric cable around her neck. The victim explained that the attack was sudden, occurring during an argument while she was seated in the kitchen. The attacker tightened the cable around her neck, causing both parties to lose balance and fall to the ground. The attacker continued to constrict the noose around the victim's neck until a third party intervened and the attacker fled. The perpetrator, however, claimed to only have intended to threaten the victim and that he had never intended to kill her. He also claimed that he had not exerted intense pressure with the cable and pointed out the lack of strangulation injuries on the victim's neck. The victim and attacker provided vastly different accounts of the event and their defenses. The primary challenge for investigators in this instance was twofold: first, to reconstruct the sequence of events leading up to the crime; and second, to establish beyond a reasonable doubt the perpetrator's intent to kill the victim. This was particularly challenging given that the victim did not exhibit the typical indicators of strangulation, including the furrow marks around the neck. Instead, the injuries were limited to certain points on the neck. To confirm their theories and reconstruct the events leading up to the crime, investigators opted to utilize a digital dynamometer to simulate the act of strangulation.

Initial investigations

**Figure 1:**
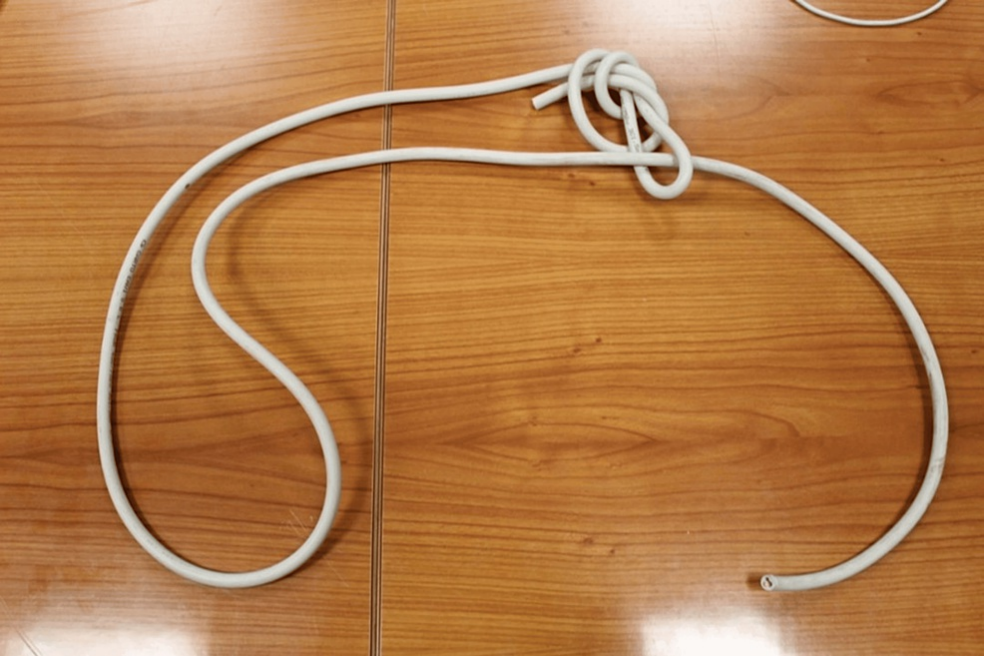
The cable that was utilized for the act on the victim

Initially, a primary collection of both the victim's and attacker's testimonies was conducted. A thorough investigation of the crime scene was carried out specifically in the house where the attack happened. All the rooms within the house were meticulously inspected. During the examination of the residence, investigators discovered the cable (Figure 1) that was utilized to strangle the woman in the man's chamber. The cable was identified as a three-pole electrical cable and comprised of a protective plastic coating with three copper cables inside, each of which is about 1 mm thick. The copper cables were further encased in additional plastic sheathings. The diameter of the cable was approximately 8 mm, and its length was around 150 cm. The cable featured a slipknot that produced a slot in which the cable could move. This design allowed the cable to be employed as a loop, which tightened its circumference when pulled. The cable was in good condition without any damage that could have altered its mechanical strength. Additionally, blood stains were detected in various areas of the cable during analysis.

Forensic investigations on the victim

**Figure 2:**
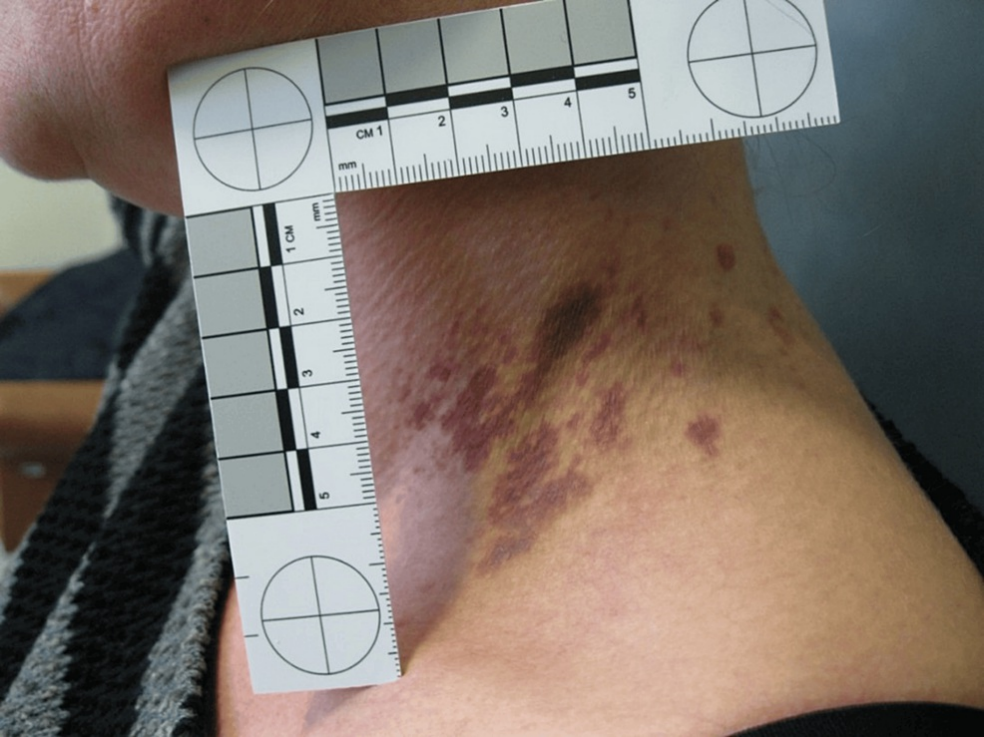
Ecchymosis on the left region of the neck

**Figure 3:**
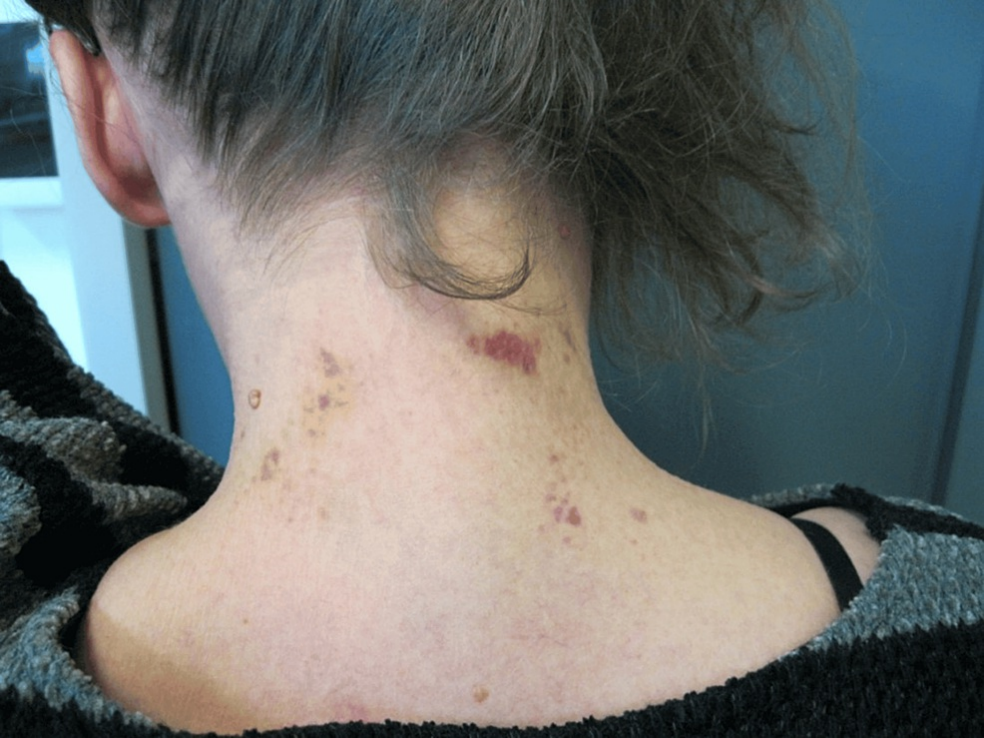
The injuries on the posterior region of the neck

The victim also underwent a careful medico-legal inspection, whereby every wound was thoroughly examined, measured, and photographed. In the second phase, the victim's anthropometric measurements were taken including height, weight, neck circumference, wrist circumference, and the transverse diameter of the hands. A complex of red-violet coloring, measuring 4x4 cm in size, was observed on the left side of the victim's neck (Figure 2). Multiple ecchymoses were also visible on the posterior side of the neck (Figure 3).

However, no injury was found on the anterior and right regions of the neck. The victim's left wrist showed extensive bruising, whereas the right wrist had a small bruised area. There was no evidence of defensive wounds on the palmar region of the victim's hands.

Analysis of the event

**Figure 4:**
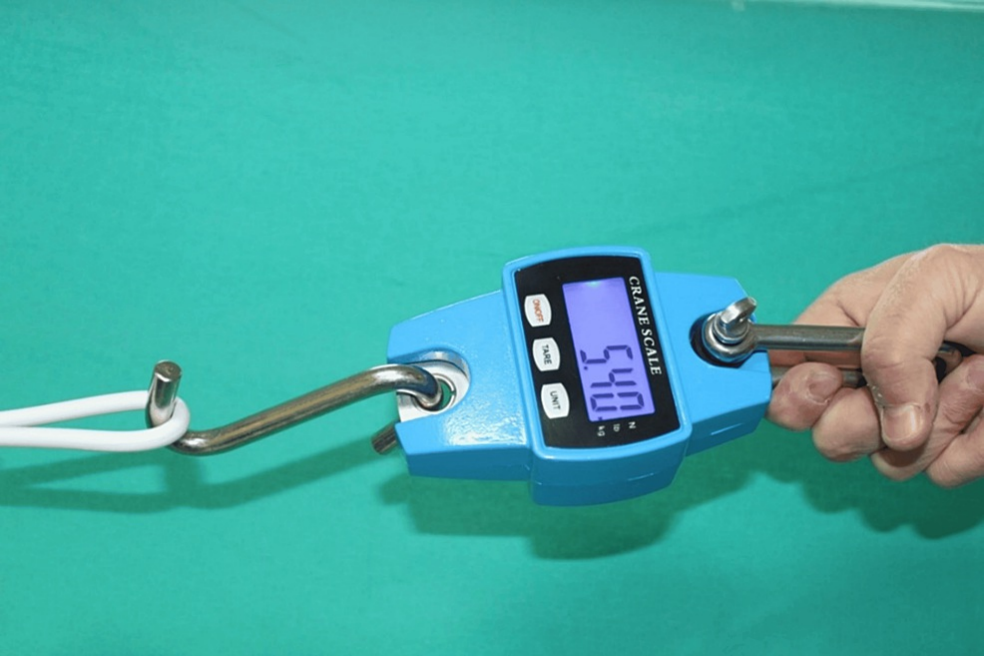
The dynamometer used in the simulations

To further analyze the dynamics and the study of force, a cable of the same length and material as the one confiscated was employed. In order to replicate a buttonhole, the cable was arranged to create a node and the end of the cable was inserted without a node, mimicking the original configuration. This newly formed circumference was then wrapped around a manikin designed with compressible soft tissue to simulate the behavior of human skin. To measure the force applied by the cable, a crane scale dynamometer was used, which is a professional digital instrument with a hook and carabiner equipped with a mechanical spring. The dynamometer had a display monitor and was capable of measuring forces up to 300 kg, providing a real-time indication of the force exerted in kilograms, Newtons, or pounds. To measure the force exerted by both men and women while pulling the cable, the dynamometer was initially tied to the free end of the cable (Figure 4).

**Figure 5:**
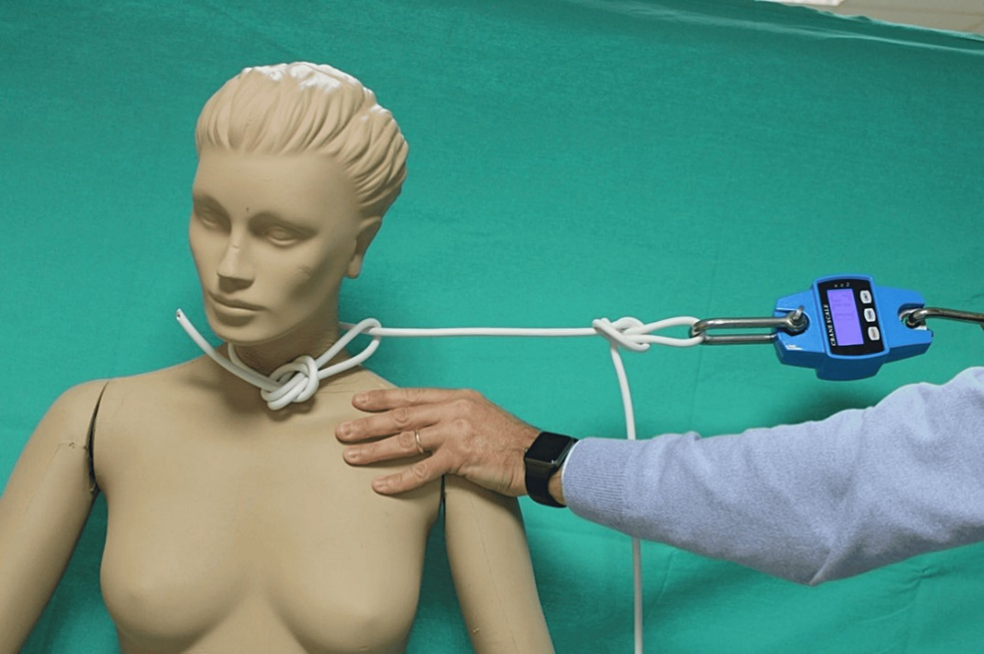
Force measurement using the dynamometer

The operators conducted tensile tests, gradually increasing the intensity until the maximum individual strength was reached. Subsequent tests were carried out to examine the possible dynamics and assess the cable-manikin interaction during the force exercise phase. Specifically, a tightening action was applied by changing the force and direction of the application with the help of a dynamometer (Figure 5). After that, the node was positioned on the lateral part of the neck and analyzed, and the injury obtained on the manikin by traction was photographed. To simulate the tightening of the noose, the investigators placed the mannequin's hands between the rope and the neck, with palms facing the face and with the back facing the face, to simulate a victim's defensive action according to two dynamics proposed by the attacker and the woman. Upon the acquisition of test results from pulling on the cable's free end, researchers made note of a decrease in the cable's circumference around the neck in proportion to the force applied; this led to a constriction of the neck's diameter.

**Figure 6:**
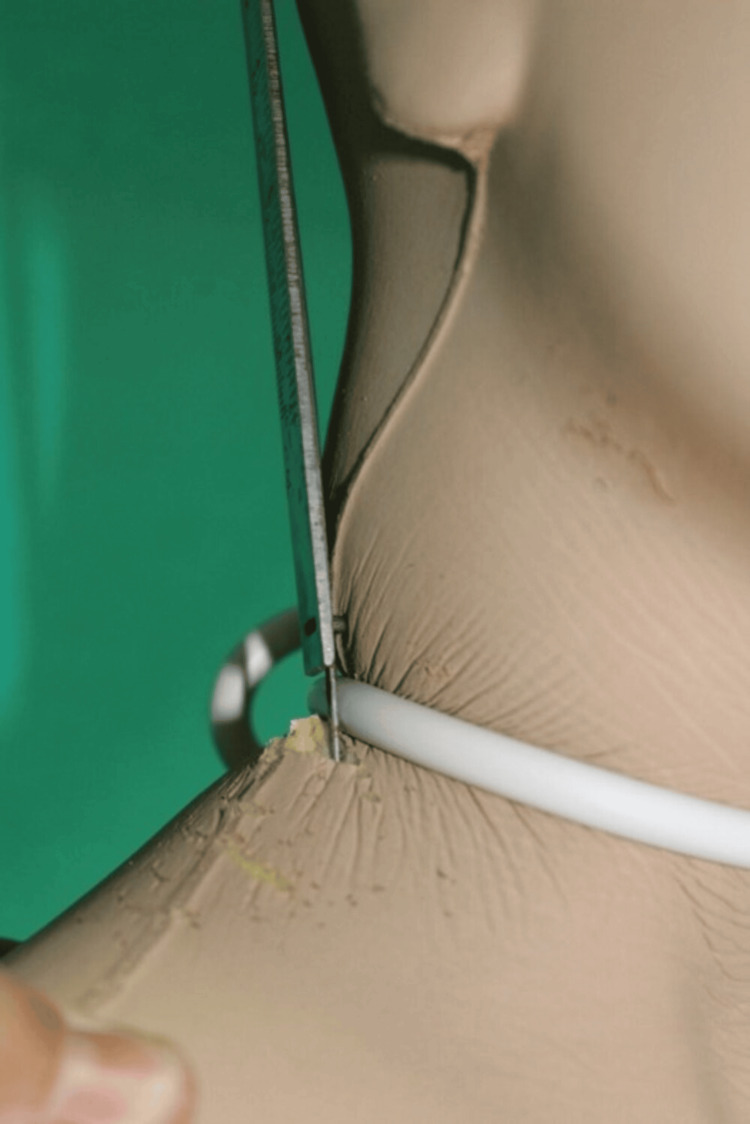
Detail of the tightening effect on the neck

The experimental model revealed that both male and female participants could easily achieve a strength value of 5 kg. With a greater amount of force applied, individuals were able to surpass force values of 20 kg. The soft surface of the manikin sustained injuries proportional to the rubbing that occurred on larger surface areas as a result of variations in the force's intensity and direction, both axially and circumferentially. Additionally, the node exhibited a significant increase in rubbing and surface changes. When the knot was positioned to one side of the neck and pulled taut, a significant increase in pressure was observed on the posterior area of the neck. This resulted in an injury that matched the victim's (Figure 6).

**Figure 7:**
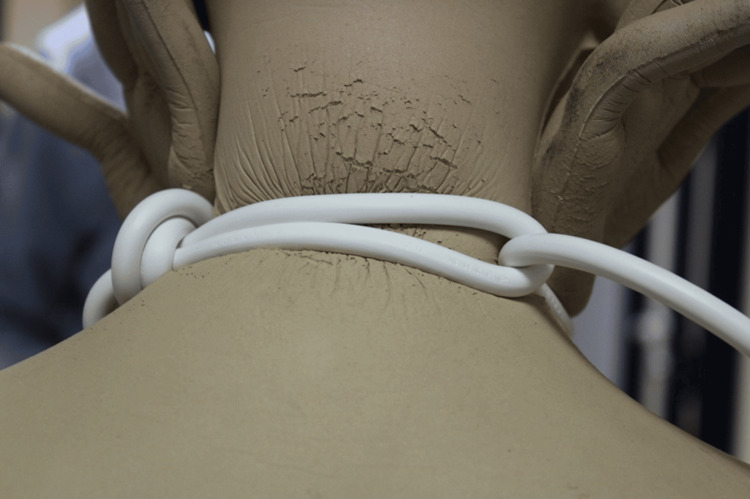
Detail of the pressure exerted on the back of the neck

**Figure 8:**
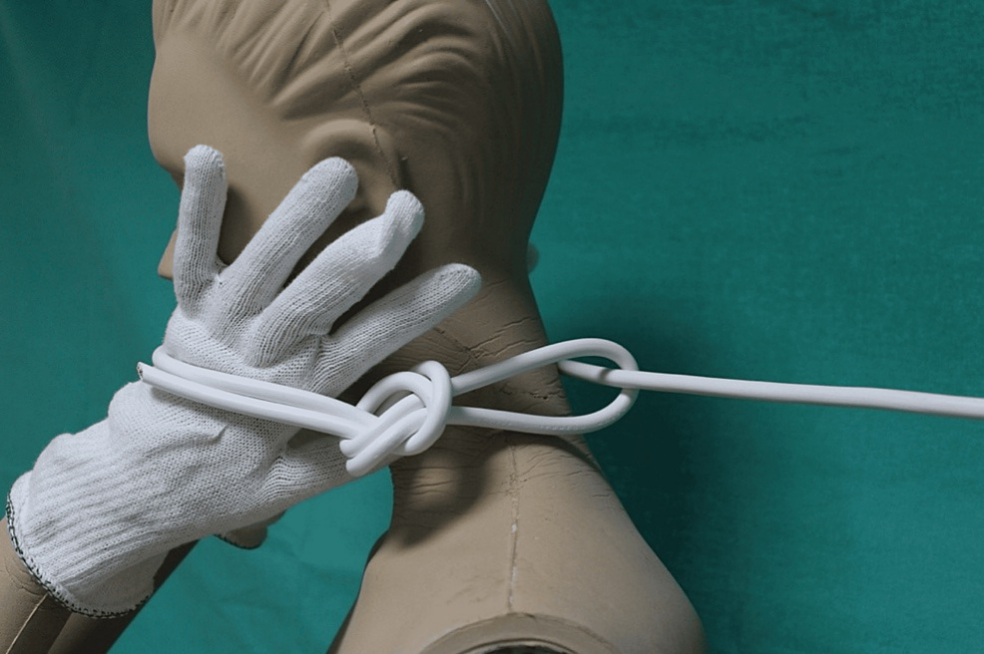
Defence simulation with the hands between the neck and the cable

**Figure 9:**
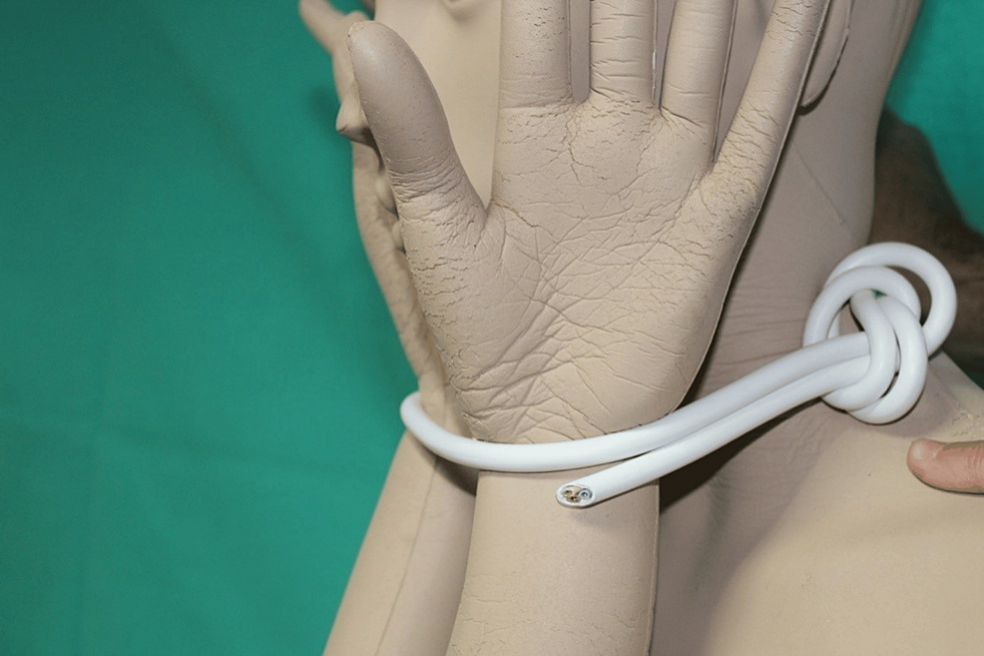
Defence simulation with the hands between the neck and the cable

By placing the mannequin's hands between the rope and the neck to simulate the tightening of the loop, it was discovered that the most significant deformations in the mannequin's fabric occurred at the knot, the back of the neck, and at the points where the cord made contact with the hands (Figures 7-9). These findings were consistent with the injuries sustained by the victim on their neck and on the back of their hands.

Our experimental model has demonstrated that when the smooth surface of a cable rubs against the skin, it can cause injuries that manifest as abrasions, grazes, or bruises. This is due to the force that is applied and the contusive action that occurs tangentially on the skin. In the current case, since the knot was situated on one side of the neck, the injuries were more pronounced in the area of the knot and the back of the neck. Using a dynamometer, we were able to simulate the positioning of the knot and the traction exerted on the cable, confirming that the knot had been positioned laterally.

The two proposed scenarios for defense dynamics involving the hands resulted in the most significant distortions occurring at the node, back of the neck, and on the hands at the points where the cable met the skin. According to our model, even if a woman were to defend herself by placing her hands under the cable, it is still possible for the cable to exert considerable force resulting in injuries limited to the posterior section of the neck.

The cable was deemed appropriate for applying pressures that exceeded 5 kilograms and had the ability to obstruct the blood flow through the neck. An adult would be able to apply such pressure with little difficulty. Additionally, if the individual being attacked loses their balance, the intensity of the downward force from their weight is combined with the vector of the upward force exerted by the assailant, intensifying the negative impact of the attack.

Due to the cable's limited surface area that made contact with the skin, the force applied caused a noticeable increase in pressure on the skin.

## Discussion

Mechanisms of strangulation

There are four mechanisms by which death from strangulation can be caused: (i) blockage of the carotid arteries; (ii) blockage or obstruction of the jugular veins; (iii) respiratory tract occlusion; and (iv) carotid sinus and glomus stimulation to trigger neurogenic reflex and cardiac arrhythmia. The methods utilized for strangulation differ depending on various personal factors, as well as the length of time the act is carried out and the level of pressure exerted on the neck.

In the realm of scientific literature, there are few experimental studies that have analyzed the minimum force values necessary to stimulate the four described mechanisms. Some pioneers in this field were Hoffman and Brouardel, who performed tests by injecting colored liquid into the neck vessels with pressure that did not exceed the maximum pressure of the blood [6-8]. They also measured the tensile forces through an experimental model, which showed a minimum value of 5 kg for carotid occlusion, 2 kg for jugular veins, and 15 kg for the trachea with upward and backward lifting of the hyoid bone [6-8]. Other authors have reported a minimum value between 2 kg and 10 kg for occluding the carotids, 2 kg for the jugular veins, and 15 kg for the trachea [5-9]. Among the more recent experimental models, Yamasaki et al. conducted a study in which they measured the minimum force required to stop the blood flow of the carotid and vertebral arteries by reproducing a pressure of 130 mmHg on the cadaver. According to the literature, a minimum force of 5 kg is required to cause occlusion of the carotid artery, while the average force to stop the blood flow in the vertebral artery corresponds to a minimum of 7.5 kg and varies significantly based on the point at which the force is applied [10].

Outcome of strangulation

The outcome of a strangulation attempt is influenced by four distinct variables, which have been identified as significant. They are: (i) the placement of the force exerted in relation to the anatomical landmarks of the neck; (ii) the measure of force's degree of strength that is commonly referred to as intensity; (iii) the length of time that a force is exerted which is referred to as the duration of force; and (iv) the surface on which force is applied that is denoted as a force application surface.

If the surface area of contact is reduced or the duration of the applied force is increased, the likelihood of the force leading to a fatality is higher, even if the force is the same. Furthermore, if a force with low intensity is applied to the right side of the neck, it can surpass the anatomical and physiological safeguards of the neck's muscles and skeleton. This means that a person with a slight build and limited strength could easily strangle someone of a larger build and greater strength [6].

Thus far, no scientific research has been conducted using a dynamometer to investigate strangulation as a means of identifying attempted murder. In our specific case, the primary challenge was determining the amount of force applied, especially considering that injuries were solely present on the left and back portion of the neck. The lack of injury on the front side of the neck was explained by the minimal force exerted by the aggressor's defense.

Upon conducting analyses of the rope and medico-legal investigations on the victim, it was observed that the victim had a large ecchymosis on the left side of the neck that was consistent with the positioning of the node in that area. The investigation also revealed that by pulling the cable, it was feasible to reduce the circumference of the loop to compress the neck. Furthermore, pulling the cable with the knot on one side of the neck resulted in a mechanism of tightening the loop at the back of the neck, which was compatible with the location of the victim's injuries. The investigators evaluated two possible scenarios where the woman could have defended herself with her forearms: either with the dorsal region of the wrists under the rope or with the volar region of the wrists under the rope. The results of the simulations showed that in both cases, it was possible to exert significant traction on the cable around the neck. Additionally, the victim's position lying on the floor exacerbated the negative effects of tightening. The means and methods employed were consistent with a strangulation attempt, accomplished by tugging on a cable with a knot situated on the left side of the neck. The experimental model ultimately concluded that the amount of force applied, irrespective of the victim's attempts at self-defense, exceeded 5 kg, and was enough to cause blood vessels in the neck to constrict [10-14]. These findings were presented in Court as scientific evidence that the manner and force of the strangulation were indeed consistent with an attempted murder [15-16].

Benefits of this experimental model

The model being experimented on boasts multiple benefits, which include the advantages of easy reproducibility and low economic costs, the opportunity to evaluate various hypotheses concerning dynamics in a comparative manner, the ability to apply and determine forces that have pre-determined magnitudes and vectors in various directions, the easy ability to compare the forces exerted with the injuries evaluated on human tissues. The use of this particular item as scientific substantiation in legal proceedings can serve to establish an individual's culpability for attempted homicide.

Disadvantages of this experimental model

The utilization of an inanimate and non-human model presents a degree of approximation in calculations, which is a notable disadvantage that must be mentioned. Additionally, it is important to highlight that this experimental model is not universally applicable to examining all forms of strangulation or asphyxiation. For example, in manual strangulation, the assailant uses their hands instead of a rope, making it increasingly difficult to replicate the dynamics and measure the value of the force applied in a precise manner with instruments such as the dynamometer. In cases of suffocation, the perpetrator may use other means to obstruct the respiratory orifices, such as their hands, pillows, envelopes, or scotch tape. As a result, this method does not allow for easy replication of the means and, therefore, hinders reliable force estimation.

## Conclusions

In our case, the dynamometer allowed us to estimate the force applied to the neck and to study the injuries caused to the tissue by analyzing potential measurable combinations in relation to the injuries suffered by the victim. The data was used in Court, providing scientific support to demonstrate the homicidal intent and the reliability of the victim's statement. The described case demonstrates the dynamometer's potential usefulness in studying forces and dynamics in other forms of violent asphyxiation, especially when a tractionable instrument, like a cord, is used to cause death, as occurs in hanging. To ensure greater precision, we suggest using this experimental model on animal cadavers. The application of experimental models with a dynamometer may prove valuable as scientific evidence in reconstructing dynamics in numerous cases of forensic interest.
